# A General Framework for Comparing Embedding Visualizations Across Class-Label Hierarchies

**DOI:** 10.1109/TVCG.2024.3456370

**Published:** 2024-12-03

**Authors:** Trevor Manz, Fritz Lekschas, Evan Greene, Greg Finak, Nils Gehlenborg

**Affiliations:** Harvard Medical School; Ozette Technologies; Ozette Technologies; Ozette Technologies; Harvard Medical School

**Keywords:** visualization, comparison, high-dimensional data, dimensionality reduction, embeddings

## Abstract

Projecting high-dimensional vectors into two dimensions for visualization, known as embedding visualization, facilitates perceptual reasoning and interpretation. Comparing multiple embedding visualizations drives decision-making in many domains, but traditional comparison methods are limited by a reliance on direct point correspondences. This requirement precludes comparisons without point correspondences, such as two different datasets of annotated images, and fails to capture meaningful higher-level relationships among point groups. To address these shortcomings, we propose a general framework for comparing embedding visualizations based on shared class labels rather than individual points. Our approach partitions points into regions corresponding to three key class concepts—confusion, neighborhood, and relative size—to characterize intra- and inter-class relationships. Informed by a preliminary user study, we implemented our framework using perceptual neighborhood graphs to define these regions and introduced metrics to quantify each concept. We demonstrate the generality of our framework with usage scenarios from machine learning and single-cell biology, highlighting our metrics’ ability to draw insightful comparisons across label hierarchies. To assess the effectiveness of our approach, we conducted an evaluation study with five machine learning researchers and six single-cell biologists using an interactive and scalable prototype built with Python, JavaScript, and Rust. Our metrics enable more structured comparisons through visual guidance and increased participants’ confidence in their findings.

## 1 Introduction

Dimensionality reduction (DR) techniques are used extensively in high-dimensional data analysis, generating lower-dimensional embeddings for tasks such as data exploration [5,18,66], data quality assessment [17, 63], and model optimization [22, 30]. In this context, data points are commonly projected to two dimensions to create a scatter plot for visual inspection. The goal is to represent the high-dimensional data structure to enable effective perceptual reasoning [3, 26, 27, 48]. For this work, we only consider these 2D scatter plots, hereafter referred to as *embedding visualizations*. For example, a researcher might compare the distribution of words like “doctor” and “nurse” in two sentence embedding visualizations generated from news articles (called model-driven comparison) to identify potential gender bias in how different professions are portrayed. Or, a biologist may compare the presence or absence of specific cell types between two embedding visualizations from healthy and diseased tissue datasets (data-driven comparisons) to identify disease markers.

Such comparisons are widespread and drive decision-making in various domains [5,8,18,19,31,40,67], yet existing methods for comparing embedding visualizations face limitations due to their dependence on direct correspondences between data points [8,23,60]. This assumption is invalid in many comparison scenarios, such as comparing embedding visualizations of different datasets, and moreover, it fails to capture higher-level relationships between groups of points [16]. Consequently, there are scenarios where current comparison metrics and visual perception are at odds, like in Figure 2 where point-based metrics suggest substantial differences between the two embedding visualizations, yet perceptually, the two visualizations look almost identical.

How can we facilitate more effective and systematic comparisons of these complex 2D scatters? The key challenge lies in establishing meaningful relationships between points in different views. The shared data in model-driven scenarios allows points to be directly compared [8, 23], whereas data-driven scenarios often require higher-level correspondences through shared class labels, given the uniqueness of points in each dataset [5, 13]. We refer to the comparison of embedding visualizations based on common class labels as *class-based comparison*.

Classes provide a natural and flexible lens for comparing embedding visualizations. They enable tasks like identifying, evaluating, and relating clusters at different levels of abstraction, since the same set of points can be regrouped. At the most granular level, individual points can serve as classes (i.e., direct correspondence), while at broader scales, classes group points with higher-level semantics. This flexibility allows comparisons across hierarchies of class labels. Even in scenarios with direct correspondence, class-based comparisons can provide nuanced insight into data patterns. For example, when comparing two embedding visualizations for models trained on images of handwritten digits [12], the coherence and arrangement of digit classes are arguably more informative than the precise placement of individual digits. Capturing and quantifying these class-level relationships not only benefits specific tasks [16], but also facilitates comparisons across different embedding visualizations.

We present a general framework for the structured comparison of two embedding visualizations based on shared labels. Our approach is grounded in perceptual theory [51] and focuses on relationships between classes and their internal compositions. For each class, we examine groups of points from three distinct areas, identified using modified one-reach and two-reach methods on a Delaunay graph [51], aimed at capturing inter-class relationships [15, 16]. This approach delineates a “core” region focused on within-class features, and a “context” region encompassing neighboring classes as illustrated in Figure 3.

The “core” region captures patterns of visual intermixing to facilitate tasks like class separability [15, 70]. We refer to this concept as **Confusion**, where the set of points in the “core” region is referred to as the *confusion set*. The “context” region supports relation-seeking tasks, such as ranking the nearest classes to a given reference class [16]. We refer to this concept as **Neighborhood**, where the set of points in the “context” region is referred to as the *neighborhood set*. Finally, the classes captured in the “core” and “context” regions define the “combined” region, which is used to evaluate the size of a reference class [15] relative to its neighborhood, which we refer to as **Size**.

For each concept, we define summary metrics for their respective point sets, enabling the comparison of these concepts across two embedding visualizations. For a class *C*, **Confusion** is quantified by the degree of visual overlap between *C* and others. **Neighborhood** is determined by the strength of connections between *C* and other classes. **Size** is assessed by comparing the relative size of *C* to its surrounding classes. Our metrics can be computed on the same embedding visualization for different class labels.

**Fig. 1:**
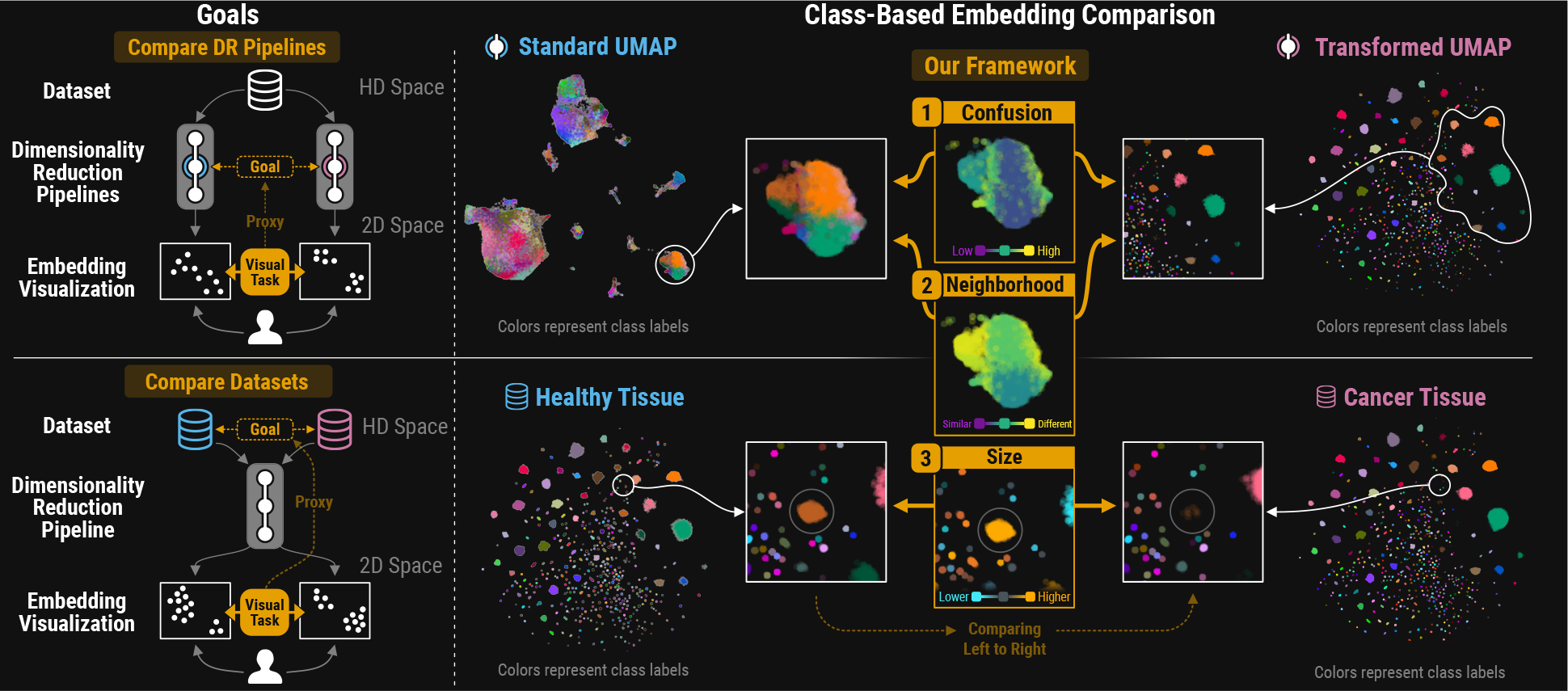
Class-Based Comparison. Left: Comparing embedding visualizations is a common downstream task, serving as a proxy for contrasting latent representations of the same (top) or different (bottom) high-dimensional datasets. Right: Our framework uses class labels to guide visual comparison of such embedding spaces based on three concepts: (1) confusion, (2) neighborhood, and (3) size. The metrics are visualized by color to reveal intra- and inter-class relationships. E.g.: the brown cluster of the tissue data (bottom left) is almost entirely missing in the cancer data (bottom right), as highlighted by the orange color in the size scatter (bottom middle).

**Fig. 2:**
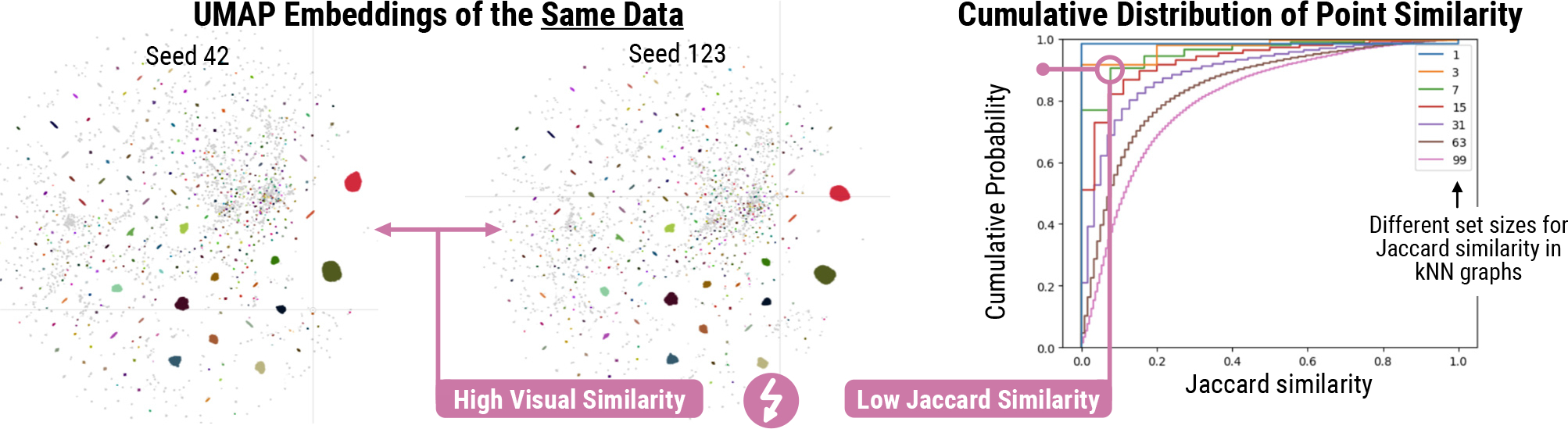
Jaccard Similarity Limitations. Comparison of two single-cell embedding visualizations using Jaccard similarity shows minimal overlap, despite only differing by randomization seed (right). With *k*=7 neighborhoods, over 90% of points have a Jaccard similarity below 0.1 (left, green line).

To inform our approach, we conducted a formative crowdsourced within-subject study to understand how people perceive class **Confusion** and **Neighborhood** stability. Using these insights and our conceptual framework, we developed an interactive embedding visualization comparison tool for Jupyter notebooks. Our prototype (Figure 1, right) visualizes the **Confusion**, **Neighborhood**, and **Size** metrics with point color in the 2D scatter plots, enabling the comparison of two embedding visualizations and revealing class-based relationships.

We applied our framework across model-driven and data-driven usage scenarios in computer vision, natural language processing, and single-cell biology, showcasing its wide applicability and ability to uncover relationships overlooked by existing methods. Through an evaluation with six single-cell biologists and five machine learning scientists, we assessed the usefulness of our framework and metrics. Our metrics increase confidence in the insights derived from comparing two embedding visualizations, and participants made more concrete and specific observations with fewer interactions. Our open-source prototype is available on GitHub (https://github.com/OzetteTech/comparative-embedding-visualization) and PyPI as cev.

## 2 Related Work

### 2.1 Evaluation of Embedding Visualizations

DR embeddings can remain high-dimensional, which complicates direct visualization. While various methods exist for visualizing high-dimensional data [38], vectors are commonly projected into 2D [48,68] or 3D [61] scatters for visual analysis. This is a common step even if the overall DR pipeline has a different goal (e.g., feature engineering).

DR inherently introduces distortions, affecting the accuracy of the lower-dimensional representation. A key concern is characterizing these errors [7, 48]. Distortion types primarily focus on point-point changes [3], either with pairwise distances between points [3] or using the overlap of nearest neighbors between spaces [48]. To address these issues, visualization techniques have been developed to aid in the analysis of embedding distortions [25, 26, 35, 48, 49].

Quality measures assess distortions at different scales. Most measures focus on relationships between individual points, including the global data structure [1, 29] and the preservation of local neighborhoods [32, 35, 47, 59]. Jeon et al., extend these pointwise concepts to clusters, introducing measures that characterize cluster-level distortions and more accurately represent inter-class relationships [16, 26].

Our work focuses on comparing two distinct 2D scatter plots, rather than evaluating the quality or reliability of embeddings. Inspired by distortion measures, our framework relates these spaces without requiring identical data points. We define class-level concepts that can be measured and compared for separate datasets. **Confusion** and **Neighborhood** in our framework are most similar to class-level reliability distortion types [26]. However, our framework is designed to operate on distinct datasets where the presence or absence of inter-class relationships carries a different meaning.

### 2.2 Visual Quality Measures and Tasks with Labeled Point Clouds

Class labels inform downstream analysis with embedding visualizations [5, 15, 16, 19, 28, 36, 56], both computationally (distortion measures) [26] or perceptually through visual cluster analysis [16, 55–57] or class separation measures [4, 6, 55]. Several quantitative metrics assess spatial characteristics of these embeddings, ranging from a scalar value for the entire embedding [14] to more local, pointwise neighborhoods [44].

Metrics can diverge from human perception of cluster structure [36, 58]. Separation measures evaluate class clustering in embeddings (cluster-label matching) [28, 70], assessed by cluster-validation measures [39, 54]. Efforts aim to align these measures with human perception [4,6], but they can bias toward well-matched clusters regardless of data separation quality [28]. Additionally, visual clustering and class separation do not address core relation-seeking tasks with labeled data, such as “which classes are most similar to a given class?” or “rank the top-k classes that are most similar to a given class.” [15, 16]. Projection Space Explorer [13] considers inter-class relationships, allowing exploration of points, classes, and their hierarchies, but relies on prior or user-defined relations.

In this work, we introduce representations of class-level relationships to compare between embedding visualizations of distinct datasets. Our framework does not presuppose that cluster structure aligns with class labels (which is often the case with real-world data). Instead, it partitions points into regions characterizing intra- and inter-class relationships to facilitate comparison.

### 2.3 Neighborhood Graphs for Perceptual Groups

Given that the visual analysis of embedding visualizations is inherently human-centric, we base the implementation of our conceptual framework on graphical representations with perceptually meaningful attributes. The Relative Neighborhood Graph (RNG) [64] is one such representation, which simplifies Delaunay triangulations based on proximity. The Reduced Delaunay Graph (RDG) [50] further develops RNG by identifying “relatively close” groups. Peng et al. [51] proposed a model for dynamic perceptual grouping based on tolerance space theory [62], using one- and two-reach methods to depict the dot patterns’ structure more accurately. Our implementation draws inspiration from RDG and simplifies point groupings into “core” and “context” regions through an traversal inspired by Peng et al.’s two-reach approach [51]. Our formative user study (section 3) confirms its alignment with human perception.

### 2.4 Techniques for Comparing Embedding Visualizations

Several visualization systems aid in comparing embedding visualizations but primarily focus on relating the same set of points between views. Boggust et al. [8] introduced Embedding Comparator, which uses k-nearest neighbors to highlight points of comparison, featuring a global scatter plot and detailed neighborhood views. embComp [23] scores relatedness with additional metrics (distance, spread, density) and a distribution summary but limits direct set comparison. Parallel Embeddings [2] uses parallel coordinates to compare embedding visualizations, clustering edges to reduce clutter and showcase broader patterns. Emblaze [60] offers animated scatter plots for interactive comparison within Jupyter notebooks but lacks support for distinct datasets with corresponding classes. Polyphony [10] merges interactive visualization of single-cell latent representations to aid human-driven cell annotation about an “anchor” embedding, but its primary design focuses on cell type annotation, leaving a gap in tools for comparing annotated cell types between groups.

Our framework is tailored for comparing embedding visualizations of both identical and distinct datasets using shared class labels, focusing on broader group patterns rather than individual object relationships (section 8). This design choice complements and addresses the limitations of existing systems, facilitating comparisons of distinct datasets as well as new usage scenarios for model evaluation.

## 3 Approach: A Framework for Comparison

Our visual analysis methodology is guided by a conceptual framework for comparing two embedding visualizations with corresponding classes. We divide the points into two distinct regions (“core” and “context”) to construct representations of intra- and inter-class relationships, tailored for embedding visualization tasks [16] and downstream comparison.

**Fig. 3:**
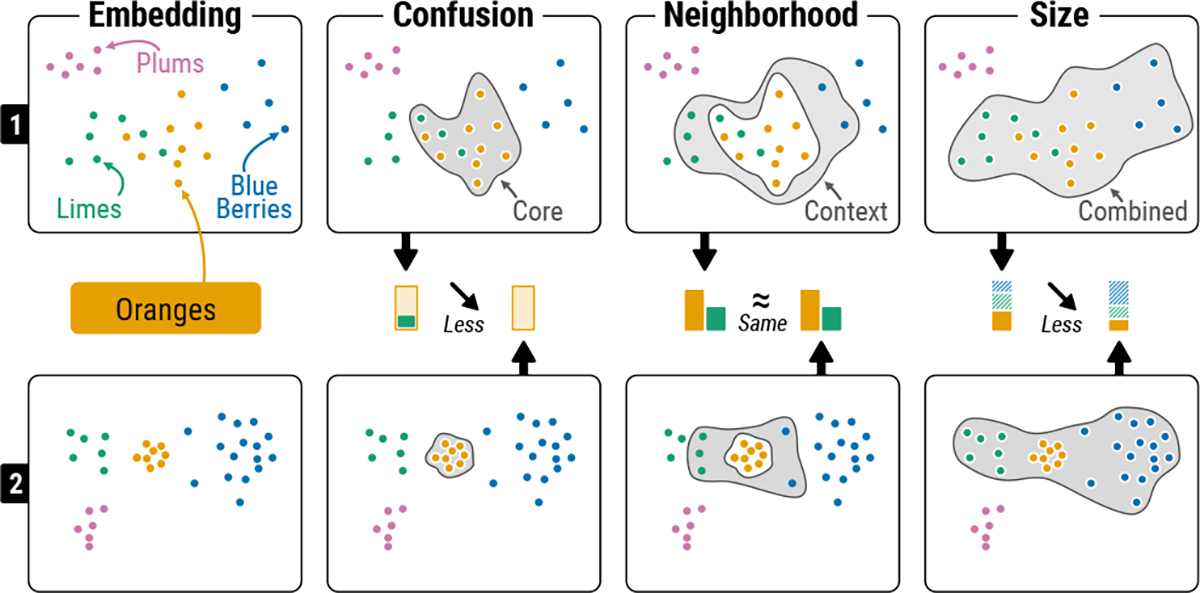
Conceptual Approach of our Framework. Two hypothetical embeddings of fruit images (rows 1 and 2) are compared using our framework. Oranges serve as the reference class. Points within each embedding are categorized into “core,” “context,” and “combined” regions (light gray area) to enable comparison of **Confusion**, **Neighborhood**, and **Size** concepts. Each region is summarized numerically, as illustrated by the bar charts between the two rows. For example, the first embedding visualization has lime images appear in orange’s “core” region, resulting in a non-zero **Confusion**, absent in the second embedding. However, in both embeddings, three lime and two blueberry images are contained in orange’s “context” region, resulting in an identical **Neighborhood**. Despite the similar absolute sizes of the orange class, the increased blueberry presence leads to a relatively smaller **Size** value compared to the first.

Our metrics (defined in section 5) summarize the composition of these regions into three key concepts: **Confusion**, **Neighborhood**, and **Size** (illustrated between the top and bottom row in Figure 3). These concepts serve as the foundation for comparing two embedding visualizations with shared class labels.

### Confusion:

For each class, its “core” region includes all its points and points from other classes with similar distributions, allowing measurement of class overlap (i.e., within-class relationships [15, 70]). Analyzing the degree of visual intermixing provides valuable insights. For instance, in Figure 3, oranges and limes overlap in the top embedding visualization but not in the bottom. Measuring this overlap is particularly useful when exploring large-scale, dense point clouds, where superimposed points can make perceptual reasoning challenging.

We define this concept of visual intermixing as the **Confusion** of a class, which accounts for the degree of visual class intermixing and the composition of the confused classes. Importantly, unlike many class separability measures, we do not assume or verify an underlying cluster structure but simply assess the extent of **Confusion** and its variation between two embedding visualizations.

Comparing **Confusion** reveals differences in data distributions, with lower confusion indicating better alignment with class labels. For different DR pipelines, variation in **Confusion** helps explain downstream model behaviors, such as classification. When comparing datasets, reduced **Confusion** suggests more well-resolved classes.

### Neighborhood:

The “context” region focuses on inter-class relationships, quantifying the consistency and changes in these relationships. It constructs a representation of the class neighborhood, weighting the influence of neighboring classes as a continuous vector. For instance, while the oranges class in the top embedding visualization in Figure 3 is more confused, the **Neighborhood** of the oranges remains unchanged as the composition of neighboring classes is the same in both.

We define this concept as the **Neighborhood** of a class. Assessing the stability and consistency of **Neighborhood** across embedding visualizations is crucial in contexts where understanding inter-class relationships is important, such as recommendation systems. When comparing different DR pipelines, minimal changes in neighborhood composition suggest that the class structure is stable, implying consistent downstream model behavior. Conversely, stable neighborhood relationships between two different datasets indicate that the relative distributions of classes are preserved, which is important for ensuring the transferability of insights across different datasets.

### Size:

Size denotes the proportional composition of a class relative to its class neighborhood. By contrasting the adjusted frequencies for each class in a pair of embedding visualizations, we identify those that undergo relative changes in size. Observing size changes highlights classes in flux and reveals broader trends in how similarly embedded classes vary between datasets. The adjusted frequencies of a class are normalized using the expanded “core” and “context” regions, or “combined” region. For smaller combined regions, this normalization offers insight into a class’s relative size compared to its immediate neighborhood. Conversely, for larger combined regions which encompass the rest of the embedding visualization, the relative size indicates shifts in the class’s absolute size. **Size** is thus related to confusion and neighborhood dynamics because less stable and more confused neighborhoods result in larger combined regions.

Relative size differences are useful for comparing different datasets or facets of the same dataset. It helps to identify class imbalances or biases, providing an understanding of data representation issues. For example, faceting news article embedding visualizations by year could reveal shifts in media focus, highlighting evolving societal trends.

Our framework bridges traditional clustering and classification concepts, using distinct regions to characterize embedding visualizations. Importantly, this approach considers all three concepts to be related, meaning the definition of the “core” region impacts the “context” region, which in turn influences the “combined” region. This integration ensures each concept uniquely measures properties while avoiding the confounding factors that arise when these concepts are considered in isolation. We propose a sequential application of the three concepts (**Confusion** → **Neighborhood** → **Size**). Understanding the level of confusion is critical for interpreting neighborhood stability and relative size differences. For instance, unstable neighborhoods with low confusion might indicate a shift in a class’s data distribution, while high confusion with unstable neighborhoods suggests high similarity between multiple class distributions, complicating conclusions about individual shifts. Despite this integration, our framework remains versatile and is not tied to any specific method for defining the “core,” “context,” and “combined” regions. The algorithms for partitioning points into these regions and summarizing them are designed to be extensible beyond our initial prototype.

## 4 Motivating Use Case: Single-Cell Biology

In single-cell biology, researchers analyze millions of cells to understand cell populations and their role in health and disease. For instance, Mair et al. [41] examine protein expression in healthy versus cancerous tissues to correlate these populations with clinical or biological features. Embedding visualizations assist in analyzing these populations. To improve the visualization of computationally identified cell populations, Greene et al. [19, 20] developed a data transformation, as shown in Figure 4.1 Middle. This transformation [19] effectively segregates cell populations compared to the untransformed data embedding (Figure 4.1 Left).

Beyond a first glance, we wonder which intermixed cell populations were separated by the transformation and whether any remain distinct. Additionally, we consider how the transformation affects the overall neighborhood structure of the embedding visualizations. Specifically, we would like to know if cell populations that are neighbors before transformation remain so afterwards. Finally, we would also like to understand how the embedding visualization properties change if we incorporate more proteins in the cell population discovery, which can result in the discovery of more specific and rare cell populations.

Answering these questions is difficult and time-consuming with large embedding visualizations. Common, visually dominant cell populations can overshadow rarer ones [24], which are often more interesting to immunologists because they might indicate unique biological behaviors. **Confusion** and **Neighborhood** guide our visual attention to cell populations of potential interest.

For instance, comparing **Confusion** and **Neighborhood** of the untransformed against the transformed UMAP embedding visualization (Figure 4.1 Left vs Middle) confirms our high-level assessment that the untransformed UMAP is more intermixed, indicated by color gradients from dark purple (unconfused) to bright yellow (confused). However, **Confusion** reveals variation in the extent to which individual cell populations overlap with others. For instance, the large cluster representing T helper cells (i.e., CD3+ and CD4+ cells) in the untransformed UMAP mixes cells with CD27 expression (purple points), without CD27 expression (gray-green points), and with CD45RA expression (gray-yellow points). In contrast, the transformed UMAP separates these cell populations (Figure 4.1.1).

Do the separated cell populations remain consistent across embedding visualizations, or has the transformation altered their relationships? In the **Neighborhood** visualization, point colors indicate neighborhood similarity, ranging from dark purple (similar neighborhood) to bright yellow (dissimilar neighborhood). The neighborhood around the T helper cell cluster (Figure 4.1.1) shows minimal change. Despite the visual separation of cell populations in the transformed UMAP, the neighborhood of the T helper cells remains stable compared to the untransformed.

With increased confidence in the data transformation’s ability to reduce visual mixing of cell populations while maintaining stable neighborhoods, we compared the healthy tissue against a cancer tissue dataset (Figure 4.1 Middle vs Right). Applying the data transformation on both datasets aligns the embeddings (see Greene et al. [19, 20] for details) and enables us to examine the relative cell population size differences using **Size**. Differences are color-coded from sky blue (smaller than reference) to bright yellow (larger than reference). Overall, we observe that the cell populations of the cancer tissue are larger (Figure 4.1 Right Bottom Size: the majority of points are yellowish). However, there is a smaller set of activated cytotoxic T cell populations (Figure 4.1.2) that is more abundant in the healthy tissue. Identifying this pattern without guidance is challenging. Biologically, this could suggest that the cancer tissue has fewer tumor-infiltrating lymphocytes, classifying it as immunologically “cold.”

Initially, we used six proteins for classification, limiting the ability to identify rare cell populations. By incorporating an additional twelve proteins, we refine our analysis (Figure 1, right). Since our framework and metrics can be applied across class hierarchies, we repeat the same comparisons using the same datasets except with more specific cell populations. Focusing on Figure 1 model-driven comparison (top), we see that the classes in the untransformed are now much more intermixed compared to the transformed. This indicates that UMAP [45] alone is not able to visually resolve highly-specific cell populations. The most well-separated cell populations in the untransformed embedding are B cells (Figure 1.1 and Figure S1.2 Confusion Set: Circled Area vs the Rest). Their neighborhood stays relatively stable also (Figure 1.2 and Figure S1.2 Neighborhood Scatter: Circled Area vs the Rest). Both metrics indicate that there are fewer and less-diverse B cell populations. Shifting our focus to the comparison between the healthy and cancer tissue, we see how some cell populations dramatically change in size (Figure 1 Right Bottom). For instance, as highlighted in Figure 1.3, the terracotta-colored class (a specific B cell population) nearly disappears in the cancer tissue, as indicated by the bright orange color in the central **Size** scatter. These shifts are immediately apparent in the **Size** visualization.

In summary, our framework enhances comparison of embedding visualizations by analyzing **Confusion**, **Neighborhood**, and **Size** at the class level, addressing the challenge of missing point correspondences. Visualizing these metrics enables rapid identification of actionable insights, improving data interpretation.

## 5 Methods: Similarity Measures

We realize our conceptual framework on a Delaunay triangulation of the embedding visualization (Figure 5), using a variation of Peng et al.’s two-reach approach [51] to define three regions per class. For each class, we use a two-step process: first, identifying the “core” region through a set number of hops on the Delaunay graph, then defining the “context” by additional hops from the “core.” We detail this process and our summary metrics for the “core,” “context,” and “combined” regions in subsequent sections. Special edge cases are discussed in Section S3.

### 5.1 User Study: Assessing Human Perception of Confusion and Neighborhood

We conducted a preliminary user study with 100 participants to assess the perception of confusion (Task 1) and neighborhood (Task 2), guiding our implementation to match human perception. Below, we summarize the tasks and findings (see Section S1 for details).

In the first task, participants rated the intermixing between two point clouds (Figure 6, Task 1). We initially found low to moderate agreement among participants, setting an upper limit for predicting perception of confusion with our metric. Nevertheless, transitioning from an unweighted one-hop search to integrating boundary edge lengths and distance thresholds to define a “core” region improved correlation of our confusion metric with human perception, refining our approach.

In the second task, participants identified neighboring classes to a central class (Figure 6, Task 2). Initial analysis based on exact neighbor matches showed low to moderate agreement. However, considering the frequency of each neighbor’s identification revealed higher consensus, indicating that neighbor presence is better represented as a continuous vector rather than a binary condition. This insight led us to evaluate class neighbor connectivity as a continuous value. We refined our representation by normalizing boundary connection strengths based on their empirical distribution across the embedding, capturing the relative nature of determining local neighbors within the broader embedding context.

### 5.2 Definitions

We introduce formal definitions for our **Confusion**, **Neighborhood**, and **Size** metrics, based on an understanding of human perception from these preliminary user studies.

#### 5.2.1 Confusion Metric

The class confusion metric quantifies the extent to which points from a given class mix with points from other classes. We first identify all graph edges which connect points i and j within the same class (C), referred to as intra-class edges, denoted as $E_{C}=e_{ij}|i,\;j\in C$. Given the pairwise distances for all intra-class edges $\left.D_{C}=d(i,j)|e_{ij}\right]inE_{C}$, we define the distance threshold $\tau =\mu_{D_{C}}+k\cdot \sigma_{D_{C}}$, where $\mu_{D_{C}}$ and $\sigma_{D_{C}}$ denote the mean and standard deviation of the intra-class distances. This thresholding accounts for Delaunay’s tendency to connect distant points which are not perceptually reasonable [51]. Here, k, a user-adjustable parameter, is set to 3 based on its effectiveness in our experiments. Increasing k enforces stricter criteria for the “core” region. We then traverse the graph for h hops (default h=1), pruning inter-class edges that exceed τ. The “core” region $R_{\mathrm{core}}$ is then defined:

$$R_{\mathrm{core}}=\underset{v\in V_{C}}{⋃}𝒯(v,h,\tau )$$

where $V_{C}$ represents the vertices in class C, and 𝒯(v,h,τ) is the set of vertices traversed from v within h hops, constrained by the distance threshold τ. We compare the summaries of these sets, such as the proportion of class C points versus others, rather than the sets themselves, allowing for comparisons even if they are of different sizes.

#### 5.2.2 Neighborhood Metric

The neighborhood metric scores inter-class connections in the graph, scoring the likelihood of other classes being neighbors. We define the “context” region $R_{\mathrm{context}}$:

$$R_{\mathrm{context}}=(\underset{v\in V_{C}}{⋃}\;𝒯(v,h+1,\infty ))\backslash R_{\mathrm{core}}$$

Vertices reachable from v within h+1 hops, without distance constraints, define the context region $R_{\mathrm{context}}$ after subtracting the core region $R_{\mathrm{core}}$. Boundary edges connecting $V_{C}$ to $R_{\mathrm{context}}$ define the neighborhood scores. The likelihood that class X is a neighbor to class C, denoted by the connectivity $\kappa_{C\to X}$, is derived from the number and length of boundary edges connecting C to X in $R_{\mathrm{context}}$. The score is the quantile from the empirical distributions across all classes. Class neighborhoods are thus represented as a continuous vector.

#### 5.2.3 Size Metric

Given the neighborhood representation, we define the size metric by multiplying this continuous vector $\kappa_{C}$ with the relative sizes of these neighboring classes, calculated using the centered log ratio of the absolute number of associated points from the $R_{\mathrm{combined}}$ set. This normalization adjusts for class size variations, ensuring the metric mirrors the true distribution. The “combined” region is defined as:

$$R_{\mathrm{combined}}=\left\{v\in V|label(v)\in L_{\mathrm{core}}\cup L_{\mathrm{context}}\right\}$$

where label(v) is the class label of vertex v, and $L_{\mathrm{core}}$ and $L_{\mathrm{context}}$ are the sets of class labels in the core and context regions, respectively.

## 6 Prototype

We developed a hybrid Rust, Python and JavaScript prototype for interactive comparison of embedding visualizations in Jupyter-like environments. This enables users to create embeddings with various Python DR libraries and compare them using our tool. To handle the scale of single-cell embeddings (section 4), we implemented core metrics in Rust for efficiency and made them accessible through Python. For interactive scatter plots, our visualization extends Jupyter Scatter [34], built on *anywidget* [42, 43] and uses regl-scatterplot [33] for GPU-accelerated rendering. The embedding comparison metrics take 0.02s for 1k points, 0.06s for 10k points, 0.35s for 100k points, and 3.3s for 1M points on an Apple M3 MacBook Pro. The system supports dynamic interactions with up to 20M points [33]. The metrics and visualization tool are open-source and published on PyPI under the package name cev. The code is available at https://github.com/OzetteTech/comparative-embedding-visualization.

## 7 Usage Scenarios

We now demonstrate our framework’s applicability to data types and domains other than single-cell embeddings (section 4).

### Comparing Embedding Methods With Unclassified Data.

Embedding visualizations are common in contexts lacking ground truth labels or with loosely structured data, making comparisons challenging. Fortunately, we can still apply our framework by establishing correspondences between points with automatic clustering methods. Assigning cluster labels from one embedding visualization to another, we compare the extent to which the 2D structure is preserved.

To illustrate, we applied our framework to embedding visualizations of headlines from news topic dataset [46]. We compared MiniLM [52] and Mpnet [53] sentence transformer embeddings visualized with t-SNE [65], using the MiniLM as the reference to derive clusters for the news articles as shared labels. The results are shown in Figure 7.1 wher the cluster labels are visualized by point colors and propagated as labels to the Mpnet embedding to establish correspondences. Using MiniLM as the reference, we analyzed **Confusion** and **Neighborhood** changes to assess cluster stability.

Overall confusion with Mpnet was low (see Figure S2), indicating well-resolved classes from MiniLM. However, neighborhood stability varied. Examining the headline text, we identified two clusters with contrasting neighborhood similarity (Figure 7.1, center). The first cluster (Figure 7.1.1) exhibits higher neighborhood similarity and contains articles on Mars and space-related topics. The second cluster (Figure 7.1.2), including articles about video games, shows substantial changes in its neighborhood. Mpnet places some video game articles differently than MiniLM, suggesting they capture different associations. The video game cluster found in MiniLM is less stable than the space-related cluster.

Establishing correspondence allows for comparisons between embedding visualizations lacking ground truth labels. In this scenario, we identified variable cluster stability when comparing MiniLM to Mpnet. This technique can be extended to compare dimensionality reduction methods (details in Section S2), emphasizing our framework’s flexibility and broad applicability.

### Faceted Comparison of Captioned Images

**Fig. 4:**
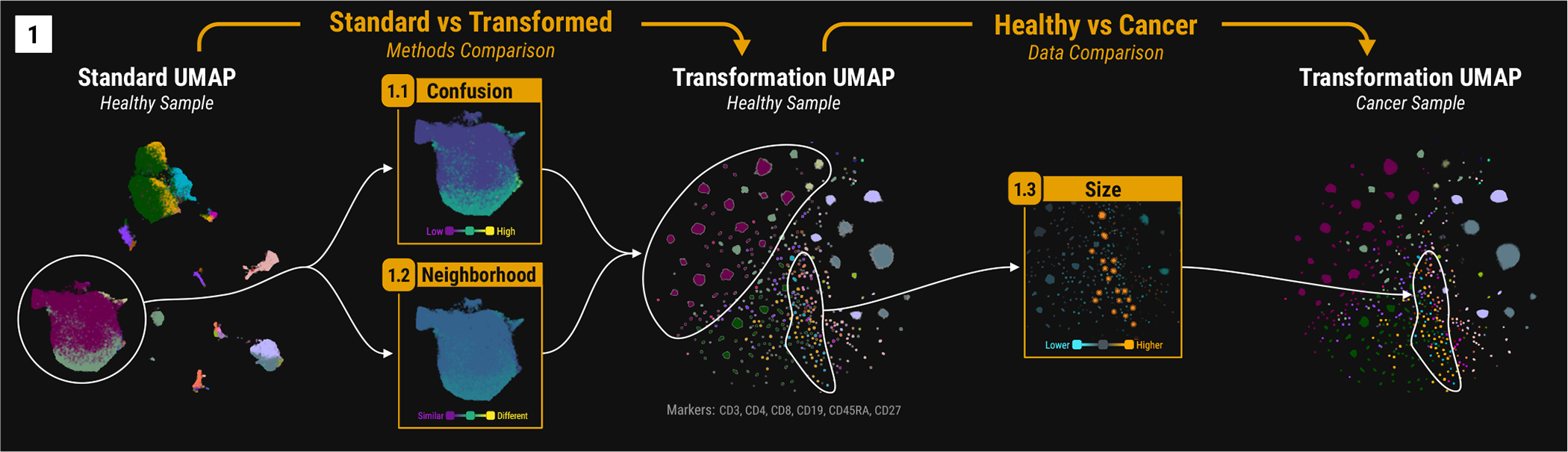
Comparing Single-Cell Data. (1) Three embedding visualizations of cell populations derived from two datasets: healthy tissue and cancer tissue, distinguished by expression of six proteins. Left: Standard UMAP of healthy tissue. Middle: Transformed UMAP of healthy tissue. Right: Transformed UMAP of cancer tissue. Between Left and Middle, the scatters visualize **Confusion** (top) and **Neighborhood** (bottom) relative to the embedding visualization in the middle. Between Middle and Right, the scatters show **Size** relative to the right (top) and middle (bottom).

**Fig. 5:**
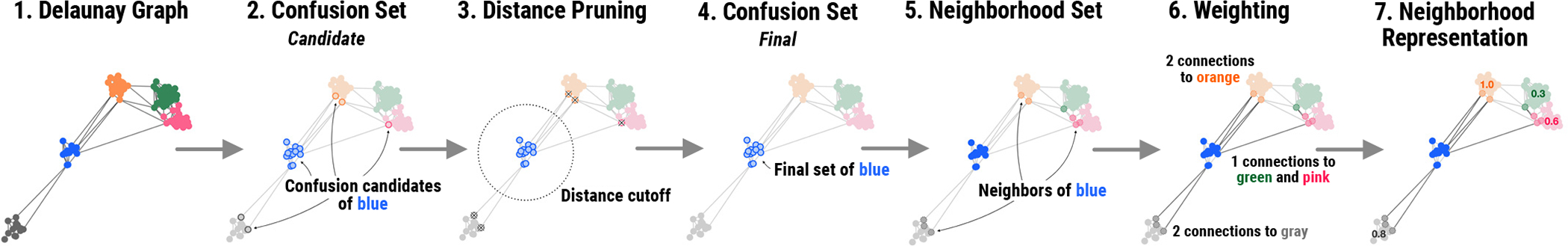
Realization of our Conceptual Metrics. The implementation of all three metrics involves summarizing regions of points derived from a Delaunay graph of the 2D embedding visualization. The illustrations demonstrate the construction of the “core,” “context,” and “combined” regions for the blue class. First, we create a candidate set of points that are h hops away from each blue point (default h=1). We then remove points whose distances exceed the τ, based on the average intra-class distance. This pruning defines the final “core” region. Next, the “context” region includes all points that are h+1 hops away from each blue point, excluding the “core” region. A continuous neighborhood representation of the points in the “context” region is created by weighting the connections and distances of the “core” to “context” boundary edges.

**Fig. 6:**
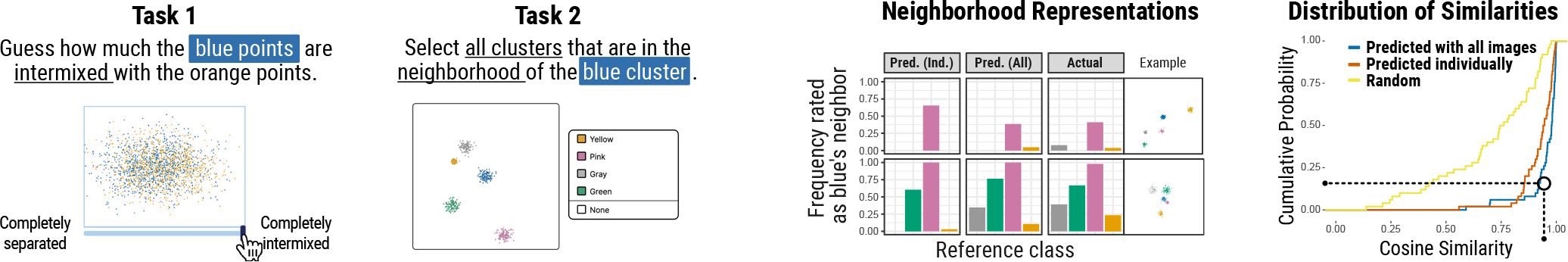
Preliminary User Study – Task Examples & Neighborhood Results. Task 1: Participants rated the level of intermixing of two point clouds. Task 2: Participants selected all classes they considered in the neighborhood of the blue class. Neighborhood representations: Predictions for individual (Pred. (Ind.)) and collective (Pred. (All)) weighting are compared to actual class frequencies, with collective weighting vs. actual for a few examples. Distribution of Similarities: ECDFs of cosine similarities between actual and predicted neighborhood frequencies in Task 2 with different weightings. For the collective weighting (blue), less than 25% exhibit similarity below 0.9; for individual weighting (red), about 50% show similarity under 0.9.

While **Size** is valuable for comparing distinct datasets (see Figure 4), its use for the same dataset might be less intuitive. Here, we present an alternative data-driven comparison approach using *faceting* to understand class size distribution across different facets of the same dataset using **Size**.

We demonstrate this approach on the COCO image dataset [37] (Figure 7.2). Briefly, we create a single embedding visualization for all image captions and facet into two groups: images with people and images without people, using ground truth annotations (Figure 7.2, upper left). Since we facet by the term “person,” we exclude it from the final class to harmonize the labels between facets. With shared correspondences we then apply the **Size** metric to compare the relative class sizes in the facets.

The faceted embedding visualization reveals variation in relative sizes of image classes, and immediately draws attention towards classes which display potential shifts. The enrichment of “surfboards” in the “with person” facet (Figure 7.2.1) suggests they are rarely photographed alone. This aligns with surfing’s focus on people riding boards, explaining their frequent co-occurrence in images. In the upper right side of the “without person” facet, there is a broad enrichment of images with animals. In this region, birds are more prevalent without people, likely reflecting natural habitat photography (Figure 7.2.2). Interestingly, the “horse” class size remains consistent (gray), suggesting frequent capture in both solo portraits and interactions with humans (Figure 7.2.3).

Facet comparisons offer a powerful tool to understand data composition and variations. This is especially true when faceting dimensions complement classification, as seen in time series. Our use of images here highlights the framework’s generalizability to various data types.

## 8 Evaluation

We conducted a user study with single-cell biologists and machine learning scientists to assess the usefulness of our proposed framework and metrics by comparing two embedding visualizations with and without our metrics.

### 8.1 Study Design and Participant Recruitment

The study followed a within-subject experiment design with one factor – *guidance*, with two levels: *unguided* and *guided*. In the unguided condition, participants compared embeddings without visual cues on focus areas. In the guided condition, they compared embeddings with two extra visualizations highlighting one of our three metrics.

We recruited 11 participants from the first author’s university, knowledgeable in machine learning or single-cell biology and experienced in using embedding visualizations, using flyers and mailing lists. They received a 25 USD gift card upon completing the study.

### 8.2 Tasks

In four tasks, participants were shown side-by-side interactive scatter plots of two embeddings, with point classes color-coded consistently across both visualizations (see Figure S3, top). For guided tasks, two additional plots highlighted our metrics (Figure S3, bottom, with dashed outline). Participants compared the embeddings and reported their findings and insights by thinking aloud. Different embeddings were used in each task to avoid memory effects.

Task 1A: Unguided Methods Comparison. Participants compared two embedding visualizations of the *same* dataset created with *different* methods (i.e., model-driven comparison [8]).

Task 1B: Unguided Datasets Comparison. Participants compared two embedding visualizations of *different* datasets created with the *same* methods (i.e., data-driven comparison [8]).

Task 2A: Guided Methods Comparison. Same as Task 1A, but included an additional scatter plot per embedding visualization displaying our **Confusion** or **Neighborhood** metric through point color. Participants could select and change the metric they visualized during exploration. The **Size** metric was excluded since the data points were consistent across both embedding visualizations.

Task 2B: Guided Datasets Comparison. Same as Task 1B, but included an additional scatter plot per embedding visualization displaying our **Size** metric. The other metrics were disabled due to their use in the previous task.

### 8.3 Data

Participants were divided into machine learning and single-cell biology groups based on their expertise, with each group receiving tailored embeddings to ensure data familiarity (detailed in Section S4). Briefly, we prepared four embedding visualization pairs of the COCO imaging dataset [37] for the machine learning group using the annotations as class labels. For model comparison tasks, we created two embedding visualizations of the same images: one using a vision model and the other using a sentence transformer on their captions. For data comparisons, we split one image embedding visualization into two facets (e.g., “with person” vs. “without person”, described in Section 7). For the single-cell biology group, we prepared two embedding visualization pairs using annotations from FAUST [19] as the shared labels. For model comparison, we prepared single-cell embeddings with and without the Greene et al. [19, 20] data transformation (see section 4 for details). For the dataset comparison, we prepared embeddings of healthy versus diseased tissue, both processed with this data transformation.

### 8.4 Procedure

Each session began with a brief introduction to embeddings and an overview of the study process. Participants completed a questionnaire on their familiarity with embeddings. We then introduced our software prototype. Participants completed four tasks in a fixed sequence with randomized embedding visualization pairs to avoid memory biases. After each task, they assessed their confidence in their findings. Before tasks 2A and 2B, we demonstrated our framework and metrics in the prototype. After all tasks, participants evaluated the framework and metrics’ usefulness.

### 8.5 Measurements

#### Confidence.

After each task, we asked “How confident are you in your insights/findings when comparing these two embeddings?”. The participants provided their answers on a 5-point Likert scale ranging from (1) “not confident at all” to (5) “very confident.” For model comparison tasks 1A and 2A we additionally asked “How confident are you in your ability to determine the level of intermixing between classes?” and “How confident are you in your ability to determine how the neighboring classes differ between the two embeddings?” using the same scale. Similarly, for data comparison tasks 1B and 2B we asked “How confident are you in your ability to determine changes in the relative sizes between classes?”. Our goal was to gauge whether our metrics enhanced participants’ confidence in their insights and conclusions with the embedding visualizations.

#### Usefulness.

After completing all tasks, we asked participants, “How helpful do you find having a framework to think about and compare embedding visualizations?” and evaluated the usefulness on a 5-point Likert scale, from “not helpful at all” (1) to “extremely helpful” (5). We also asked, “How useful is the concept of <metric> for thinking about and comparing embedding visualizations?” to evaluate each metric on the same Likert scale. Our aim was to assess the perceived usefulness of our framework and its three components.

#### Qualitative Measurements.

We also collected qualitative measurements through open-ended questions and think aloud observations. In the pre-study questionnaire, participants were shown an embedding visualization of the Fashion MNIST [71] dataset using UMAP [45] and posed the question: “When observing this embedding, what visual patterns did you focus on?”. They were then presented the same visualization alongside a second embedding visualization of the same data that used t-SNE [65] and asked, “Have you ever considered comparing two embeddings before? If so, what do you visually focus on when examining these two embeddings side by side?” Finally, we asked, “How important are the individual points to you? Are you more focused on individual data points, or the broader patterns of groups of points with the same class?”

### 8.6 Results

#### 8.6.1 Confidence

**Fig. 8:**
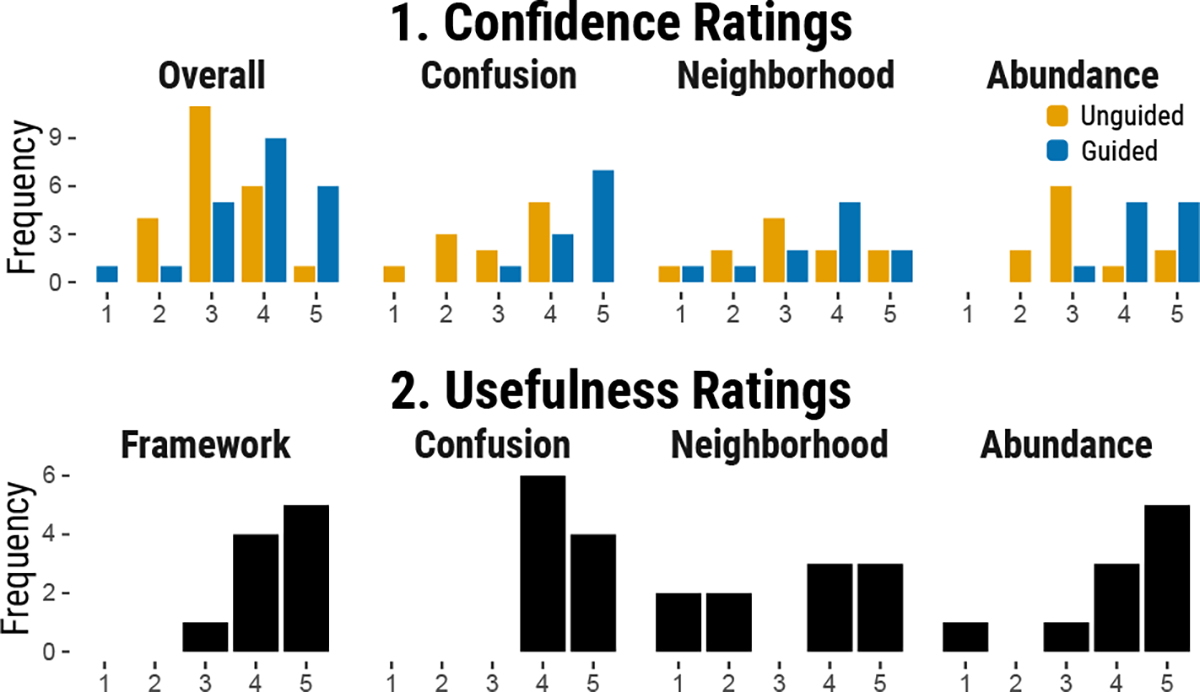
User Study Results on Confidence and Usefulness Confidence ratings are on a 5-point Likert scale, comparing unguided and guided embedding visualization tasks using our framework. The usefulness ratings are also on a 5-point Likert scale and reflect the overall perceived usefulness of our conceptual framework.

To evaluate the change in confidence when using our metrics versus unguided visual comparison of two embeddings, we compared the confidence ratings of Tasks 1A and 2A as well as 1B and 2B. The ratings distribution is shown in Figure 8. We performed ordinal logistic regression with guidance (yes/no) and focus (none, confusion, neighborhood, size) as independent variables, and ratings as the dependent variable. Using no guidance and no focus as the reference, we found that the odds of being *more* confident were 5.24 times higher (95% CI, 2.52 to 11.28) with guided comparison, a statistically significant effect (p<0.0001). Focus (confusion, neighborhood, size) had no significant effect on confidence ratings. However, the mean confidence difference between guided and unguided comparisons was strongest for confusion (M=1.36) and size (M=0.91), followed by overall (M=0.68) and neighborhood (M=0.36).

These results suggest that guided comparisons increase participants’ confidence when comparing embeddings, particularly when focusing on confusion and size metrics.

#### 8.6.2 Usefulness

To evaluate our framework’s utility, participants rated its overall usefulness and the usefulness of specific metrics. Since our tool uniquely supports class comparisons within embeddings, direct comparison with existing methods was not possible. We report the following means and standard deviations: framework usefulness M=4.4, SD=0.67; confusion metric usefulness M=4.4, SD=0.51; neighborhood metric usefulness M=3.3, SD=1.6; and size metric usefulness M=4.1, SD=1.3.

In the post-survey, participants predominantly rated our framework for comparing embedding visualizations as useful, with a mean rating of 4.4 (SD=0.67) (8). The confusion metric was favored, with a mean of 4.4 (SD=0.51); P4 and P8 noted its value in revealing inner cluster structures and understanding overlap. The neighborhood metric received mixed reviews, with a mean of 3.3 (SD=1.6). While P6 found it beneficial in scenarios with class hierarchies, P5 and P7 found it less intuitive. P2 expressed concerns with its implementation, stating, “The neighborhood calculates the similarity of the neighboring groups but without the group itself, which seems a little indirect in indicating patterns.” The size metric was praised for its utility in comparing samples, with a mean of 4.1 (SD=1.3). However, P10 expressed difficulty in grasping this concept and its use case.

In summary, participants rated our framework as useful for comparing embedding visualizations, with the confusion and size metrics being particularly appreciated, though feedback on the neighborhood metric was mixed.

#### 8.6.3 Pre-Study Questionnaire

Participants emphasized two key features when presented with the first embedding: clusters [P1-P11] and classes [P1, P2, P5-P9]. Internal attributes of clusters (size [P4, P9], shape [P4, P8], number [P1]) and classes (size [P5], number [P1]) were important to some, but most participants were interested in specific patterns that relate these features [P1-P3, P5-P6, P8, P10], such as extremes in cluster density [P3], similarities in class distribution within clusters [P2], and the relationship between clusters and class geometries [P6]. Additionally, participants frequently noted the distances between groups [P1, P3, P8, P11] and the degree of class overlap within and between clusters [P2-P4, P6-P7]. Only P5 mentioned individual points or outliers.

When comparing two embeddings, 8 out of 10 participants had prior experience. P1 approached the comparison abstractly, focusing on pattern identification and correspondence between two spaces. Others identified specific patterns between spaces: class intermixing or overlap [P1, P8, P10]; relative positioning of classes both locally [P1, P3] and globally [P3]; and the spread, density, or size of clusters and classes [P3-P4, P9-P10]. All participants prioritized groups of points with the same class over individual points, though some noted an interest in individual points for identifying outliers [P1-P2, P4, P9].

Overall, the responses show that participants focused primarily on clusters and classes, their relationships, and patterns within and between them, with less emphasis on individual points or outliers.

#### 8.6.4 Observations From the Methods Comparison Tasks

##### Unguided.

Some participants had a clear task in mind from the start, while others struggled to begin. P1 quickly expressed a goal, “I’m just trying to look for concept overlap,” and soon identified that annotation X often overlaps with X+Person. P3 also noticed this pattern. In contrast, several participants felt overwhelmed by the number of classes and were unsure where to start [P2-P3, P5-P6]. When asked about overall confidence, two participants mentioned they would likely be more confident with more time using the data and tool [P5, P10].

##### Guided.

The embedding comparison was primarily informed by the metric views. Most participants concluded one embedding was more or less confused overall compared to the other [P1-P9]. The confusion metric helped some understand local structures [P2, P4, P8], noting they would have overlooked or misinterpreted details without it. P1 highlighted the metric’s insight, saying, “This is really useful, I can clearly see there is less confusion in the languages model.” The neighborhood metric was used less frequently than confusion. While some were satisfied with identifying global neighborhood differences [P1], others were curious why particular classes exhibited more or fewer changes to their neighborhood [P2]. P9 saw the conceptual value in the metric but required a deeper understanding and trust in its highlights to utilize it effectively. When asked about their exploration focus, most participants expressed primary interest in broader patterns and cluster shapes, with only occasional attention to outliers or individual data points. This consistent response indicates a preference for understanding overarching structures over specific point details.

These observations suggest that the guided metric views helped participants better understand local and global structures, while the unguided approach left some participants feeling uncertain and overwhelmed.

#### 8.6.5 Observations From the Data Comparison Tasks

##### Unguided.

Comparing embeddings of different datasets was relatively unfamiliar for machine learning scientists. Some identified similarities in the embedded spaces [P1, P10], though P10 remarked, “This isn’t that important to me. The sizes aren’t something I’m interested in.” In contrast, those working with single-cell embeddings immediately looked for size differences [P2, P4, P6]. Participants used interactive zoom and pan to analyze point densities. P2 added, “Ideally [the differences] should just jump out.” Several participants expressed concern about unknown size differences between datasets, complicating accurate assessments of relative differences [P1-P2, P5-P6].

##### Guided.

Participants experienced with single-cell embeddings were enthusiastic about the size view, commenting on its enhancement of their exploration [P2, P4, P7-P9]. P8 noted, “I notice things very quickly, much easier than before,” and P4 added, “I can immediately find differences and examine them.” In contrast, machine learning participants [P1, P3, P10] found the size view unfamiliar and were unsure how to derive meaningful insights. P1 commented, “Groups do jump out; I’m just not sure how to interpret it.” However, P5 identified that giraffe and elephant are enriched in images without people.

Together, these observations suggest the guided size view enhanced exploration for participants familiar with single-cell embeddings, while those with a machine learning background found it less intuitive.

## 9 Discussion

Our work introduces a framework and prototype system for comparing embedding visualizations. Unlike existing methods that focus on individual points, we measure and compare relationships between groups of points using shared class labels.

This approach enables us to support both model-driven and data-driven embedding comparisons, including those between distinct datasets—a previously unaddressed use case. It generalizes to many data types and applications, evidenced by three usage scenarios and positive feedback from the evaluation study. Anecdotal evidence from the pre-study survey also suggests our framework also aligns with users’ natural analytical processes for comparing embedding visualizations. Participants noted class-level patterns like mixing [P2-P4, P6-P7], distance [P1, P3, P8, P11], and layout [P1, P3] as important, aligning with core concepts of our framework.

Our evaluation study provides evidence supporting the framework’s efficacy. Participants using our guided comparison prototype were more confident in their findings compared to an unguided approach. Further, anecdotal feedback suggests our approach facilitates generating specific, and actionable insights. For instance, P2 struggled to contrast relative class sizes without guidance, stating, “It seems these Myeloid cells have increased in abundance, but I’m searching for a control population to mentally normalize.” Upon seeing the **Size** view, P2 exclaimed, “This is what I wanted before!” P8 found the confusion view effective for revealing misconceptions, noting, “I found some regions I assumed were low confusion just looking at the top [class] view, but are actually intermixed. This is useful.”

Additionally, our approach addresses hierarchical structures within data, often overlooked by existing tools. It enables drawing comparisons at different levels of abstraction, such as more or less granular cell-type annotations, shown in Figure 4. Although our evaluation study focused on the general usefulness of our metrics and did not highlight this feature, several participants anticipated its value. They requested features like modifying existing class-label groupings [P1] or applying higher-level labels from a cell-type hierarchy [P4].

This work demonstrates the value of comparing embedding visualizations based on shared class labels, but further research is needed to assess the impact of our metrics and visualizations. We emphasize the generality and extensibility of our framework. Future studies can build on our implementation or introduce new methods for defining and summarizing its core regions. These studies should examine how this framework affects user insights during comparisons and define what constitutes an insight. Alternative visual representations of the metrics are also an area of open research. For example, a glyph summarizing the neighborhood representation could enhance understanding of specific inter-class relationships. Additionally, extending this approach to compare more than two embedding visualizations remains an open research question, likely requiring different methods for visualizing changes like variance.

Our approach addresses a specific gap in the broader analysis of embeddings–comparing downstream 2D representations. It should be used alongside other methods that assess embedding quality to avoid potential misuse from drawing conclusions based solely on 2D similarities and differences without understanding the reliability of the underlying embedding and DR methods [9, 11, 69]. Note the dependencies between our metrics (**Neighborhood** depends on **Confusion**, **Size** on **Neighborhood**), which can affect interpretation. For example, if two embeddings are highly confused, neighborhood changes might be hard to interpret, and size changes might be relative to all classes. Future work exploring independent comparison metrics would be beneficial.

## 10 Conclusion

In this paper, we argue that traditional methods for comparing embedding visualizations are limited by their reliance on direct correspondences between data points. By focusing on higher-level relationships through shared class labels, our approach circumvents these limitations and offers a more flexible lens for comparing two scatter-based embedding visualizations. Complementing previous work focused on the quality of lower-dimensional embedding representations, we see this as a promising first step towards more structured exploration and decision-making with multiple embeddings.

## Supplementary Material

supp2-3456370

supp1-3456370

## Figures and Tables

**Fig. 7:**
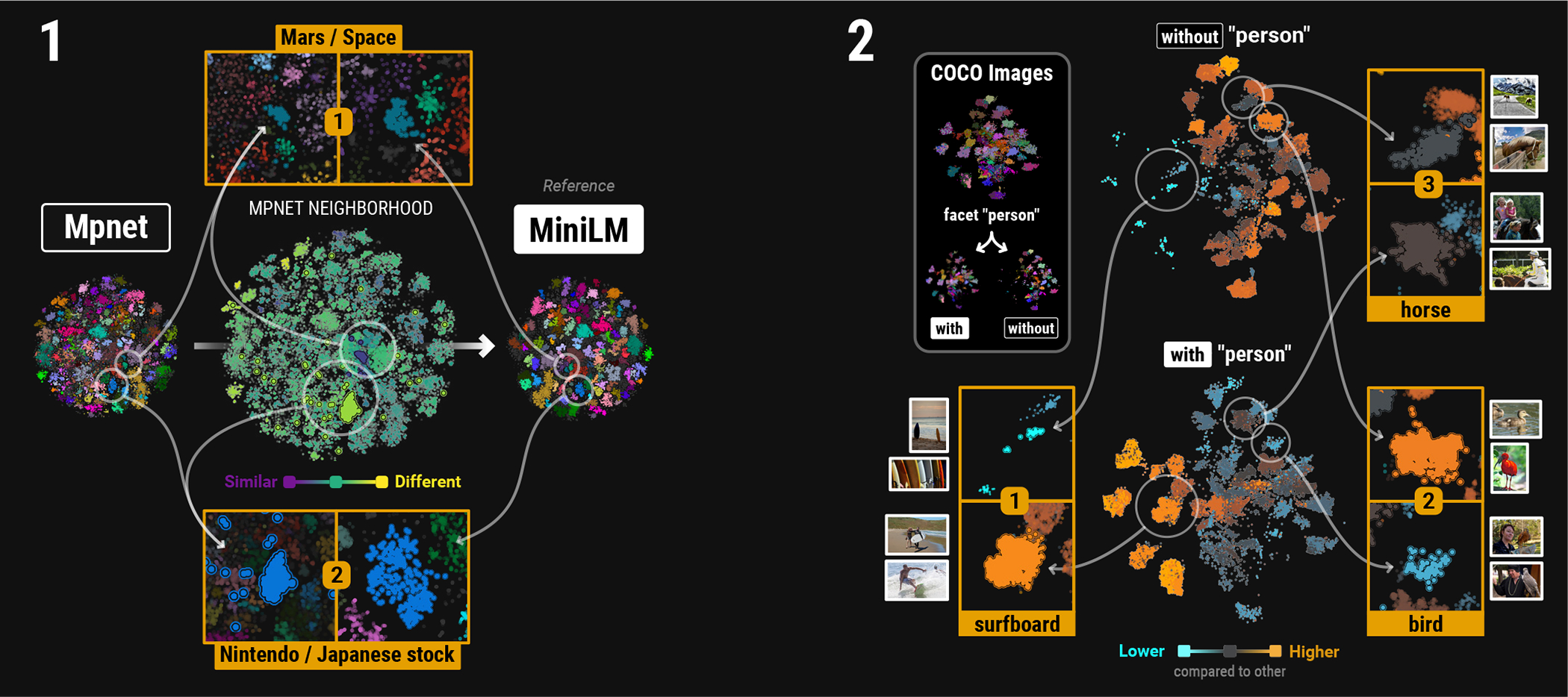
Usage scenarios Left: AG’s news headlines [21] (details in [72]), embedded with both Mpnet [53] and MiniLM [52] sentence transformer models and represented with t-SNE [65]. MiniLM is the reference used for clustering, with point colors indicating clusters and dark gray for noise. The **Neighborhood** metric view for Mpnet is centered, highlighting a high stability (1) and low stability (2) neighborhood. Right: Embedding visualization of COCO imaging dataset [37]. Image captions embedded with MiniLM and faceted into “with person” and “without person.” Each point represents an image colored by its annotation (excluding “person”). The **Size** for each facet (centered) compares the relative sizes of classes across facets, highlighting classes which display relative changes in size. Specific examples are shown, with (1) “surfboards” enriched in the “with person” facet and (2) “birds” enriched in “without person.” Notably, the (3) “horse” class size is consistent across both facets.
