# Supplementary material for: A General Framework for Comparing Embedding Visualizations Across Class-Label Hierarchies
Source: IEEE Trans Vis Comput Graph. Author manuscript; Available in PMC 2025 Mar 4. (PMC11875997; doi:10.1109/TVCG.2024.3456370)
Supplement: supp2-3456370 [file NIHMS2039886-supplement-supp2-3456370.pdf]

A General Framework for Comparing Embedding Visualizations Across Label Hierarchies

Supplementary Material

Trevor Manz
trevor_manz@g.harvard.edu
Harvard Medical School

Fritz Lekschas
fritz@ozette.com
Ozette Technologies

Evan Greene
evan@ozette.com
Ozette Technologies

Greg Finak
greg@ozette.com
Ozette Technologies

Nils Gehlenborg
nils@hms.harvard.edu
Harvard Medical School

1 Preliminary User Study: Assessing Human Perception of Confusion and Neighborhood

We conducted two within-subject user studies to characterize human perception of confusion and neighborhoods to inform our approach. The studies were crowdsourced online via the online Prolific Academic platform, where we recruited 50 participants for each task. The participants had up to 15 minutes to complete each task.

1.1 Task 1: Assessing Perceived Intermixing

The initial task was devised to evaluate participants' perception of confusion in synthetic datasets containing varying degrees of overlap between two clusters. Participants were shown 50 distinct examples containing two point clouds with various levels of intermixing. We asked participants to "guess how much the blue points are intermixed with the orange points" by placing a slider between "completely separated" and "completely intermixed", indicating the degree of intermixing of the blue points. To discourage a default rating, the drag handle was hidden until the participants clicked on the slider.

We observed moderate agreement among participants in determining the extent of intermixing between blue and orange clusters (Krippendorff's $\alpha = 0.58$), with higher consensus for low and high extremes of intermixing (less than 0.1 and greater than 0.9). A parabolic relationship between intermixing scores and variance was noted, with low variance at the extremities and increased variance for intermediate intermixing scores. This pattern suggests that participants' perceptions of confusion varied more for moderately intermingled clusters.

We investigated the correlation between the confusion identified by our algorithm and the participants' assessments, acknowledging the inherent limitation in achieving a strong correlation due to the moderate agreement among participants. Our initial implementation employed a naive breadth-first search (BFS) on the Delaunay graph for each class up to a certain distance, finding that a maximum depth of one yielded the strongest correlation with participants' assessments ($r_s = 0.57$, $p < 0.001$). To refine our method, we incorporated boundary edge lengths and determined a distance threshold in a weighted search, resulting in a slightly improved correlation ($r_s = 0.60$, $p < 0.001$) that better aligned with human perception of confusion. These findings enabled us to refine our methodology to include distances when determining what to include in the confusion set.

1.2 Task 2: Assessing Neighborhood Perception

This task aimed to assess participants' understanding of neighborhoods within spatial embeddings, using images of five distinct clusters, including a central blue cluster. Clusters were generated using a Gaussian distribution, where each cluster's points were randomly placed around a central location with a specific standard deviation, influencing point density and cluster spread. This method introduced variations in closeness, orientation, and density across clusters. Distance from the central cluster was chosen from predefined options, representing different levels of proximity. Angles were selected uniformly to vary the clusters' directional placement around the central cluster, and standard deviations were chosen to alter the clusters' densities, creating a diverse range of neighborhood scenarios. For the exact code, see `https://github.com/manzt/cev-user-study/blob/main/task2.py`. Participants were asked to identify clusters they perceived as being in the neighborhood of the central blue cluster, based on factors like spatial closeness, point density, and direct connectivity, without relying on a strict definition of "neighborhood." This approach encouraged participants to apply their judgment in evaluating complex spatial relationships.

Initially, we examined participant responses by treating each combination of neighbors as distinct, so

participants only agreed if they identified the same exact neighbors. While overall there was low to moderate agreement among participants (Krippendorff's $\alpha = 0.50$), examining individual cases and responses revealed deeper agreement than initially perceived. For each example, we analyzed the frequency in which each class was identified as a neighbor and saw that in many cases there was very high agreement across all participants for certain classes and less so for others. Consequently, we updated our approach to incorporate a more continuous view of local neighborhoods.

In this revised methodology, neighborhoods are represented as vectors of weights for each class within an embedding. In our implementation, we traverse the Delaunay graph further and score neighboring points based on the strength of their connections to the confusion region. The weight or contribution of L_B to class L_A neighborhood depends on both the number of edges from L_A to L_B outside of the confusion region, or *boundary edges*, and the length of these edges. This influence is derived from the empirical distribution of average counts and lengths of boundary edges.

When comparing the neighborhood distribution derived by our algorithm to the participants' answers, we initially observed moderate agreement, but with limitations. Specifically, our algorithm had difficulties agreeing with examples where users frequently determined no neighbors. Since users were presented with all images when identifying neighbors in the study, we adapted our approach to derive the likelihood function using boundary edges found across *all* images instead of only those within a single image. We discovered that the distribution of χ^2 distances between the predicted neighbor frequencies and frequencies observed in our study significantly improved when considering all examples rather than just the information from a single example (M = 0.11, SD = 0.096, and M = 0.23, SD = 0.173, respectively). To assess the alignment of our metric with human perception, we computed the cosine similarity between the predicted neighborhood vector and the perceived neighborhood, where the latter represents the frequencies at which participants identified each class as a neighbor. Our metric is in agreement with human perceptions, and moreover, this alignment is improved when we consider all boundary edges are factored into the construction of the likelihood.

2 Considerations on Delaunay Triangulation and Co-circularity

Our framework uses Delaunay triangulation to construct graphs from point sets. While Delaunay triangulations are generally unique, cases of co-circularity (where four or more points lie on a common circle) can result in multiple valid Delaunay graphs. We see this as a special edge case that is not going to have any impact on our proposed metrics for two reasons. First, in real-world data, co-circularity is a rare event as embedding and DR methods strive to find clusters. Second, even if it happens, it would have a noticeable impact for comparison with our metrics as we compare the summaries of our metrics. Single point differences would be averaged out for non-small classes. However, datasets with small class sizes or grid-like structures might be more susceptible to this edge case.

To verify the frequency of co-circularity, our Delaunay algorithm tracks instances of co-circularity during triangulation. We do not find a single instances in any of the embeddings presented in the usage scenarios and user study. End users may use this function to check the extent to which there may be ambiguities using our metrics.

3 Evaluating Stability Across Dimensionality Reduction Methods

Another common methods comparison can involve the dimensionality reduction methods. Since we cannot directly visualize an embedding space with more than three dimensions, we need to first reduce the dimensions down to three or (most commonly) two. Two common non-linear methods to accomplish this are t-SNE [1] and UMAP [2]. To determine whether the perceived clusters found in `minilm` are due to t-SNE, we created a third embedding using `minilm` and UMAP (Figure S2 right). As before, we propagated the classes from

minilm plus t-SNE. At a first glance, the embedding visualization with UMAP looks quite different. However, upon inspecting **Confusion** we see that most clusters are not or only mildly confused as indicated by the majority of dark purple and blue-green points. When inspecting the **Neighborhood** changes we see more changes. In particular, there is a cluster of bright yellow points located at the bottom (Figure S2.3). Selecting this cluster reveals that some of its points are located all over the UMAP embedding (Figure S2.3 right). It turns out that this is the same cluster of video game news articles that we already discovered when comparing the neighborhood stability between minilm and mpnet. This further emphasizes the instability of this clusters.

4 Evaluation Study: Data Preparation

Participants were grouped based on their expertise into machine learning or single-cell biology categories. We provided different embeddings for each group, ensuring participants had a fundamental understanding and familiarity with the data presented.

For the machine learning group, we prepared four embedding pairs of the COCO imaging dataset [3], containing richly annotated and captioned images. We classified each image in the dataset with all its associated annotations, like "person", "dog", or "airplane", yielding semantically meaningful groups of paints which can be compared in the embedding spaces. The first set of comparisons employed the vit-base-patch16-224-in21k vision transformer [4] and minilm sentence transformer [5], with dimensionality reduction via UMAP [2]. A second set used the same initial embeddings but applied UMAP and t-SNE [1] for final visualization. Data comparisons focused on contrasting facets ("with person" vs. "without person", and "with car" vs. "without car") to explore semantic groupings in the embedding spaces.

For the single-cell biology group, we prepared two embedding pairs using data from Mair et al. [6] that was clustered with FAUST [7]. For the methods comparison, we used embeddings of a tissue sample, processed once with raw data and once with annotation-transformed data [8]. Both sets were then dimensionally reduced using UMAP [2]. For the dataset comparison, we prepared embeddings of healthy tissue versus a cancer sample, both processed with annotation transformation followed by UMAP. Additionally, utilizing data from Lu et al. [9], we conducted both methods and data comparisons: the methods comparison replicated the previous embedding approach, while for data comparison, we contrasted resistor and non-resistor samples.

Supplementary Figures

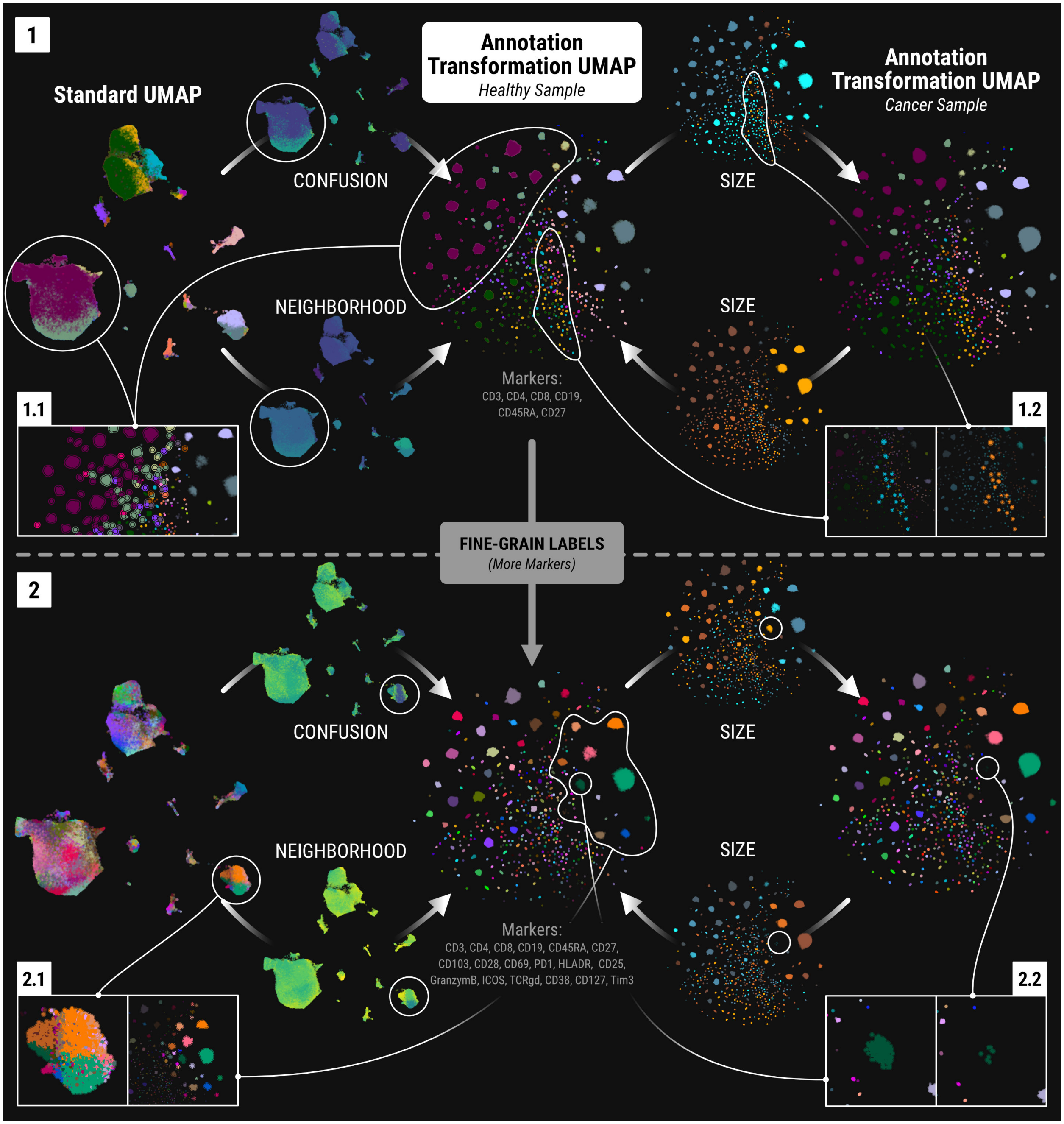

Figure S1: **Comparing Single-Cell Data.** (1) Three embeddings derived from two datasets: tissue and cancer. Six proteins were taking into account for detecting cell populations, which are visualized by the point colors. Left: A standard UMAP embedding of the protein expressions of the tissue sample. Middle: A UMAP embedding of the annotation transformed protein expressions of the tissue sample. Right: A UMAP embedding of the annotation-transformed protein expressions of the cancer sample. The two scatter plots in between Left and Middle visualize Confusion (top) and Neighborhood (bottom) relative to the embedding in the middle. The two scatter plots in between Middle and Right show Size relative to the right (top) and middle (bottom). (2) The same setup of embedding visualizations as above but this we employed fine-grain labels derived from taking into account the expressions of 18 proteins.

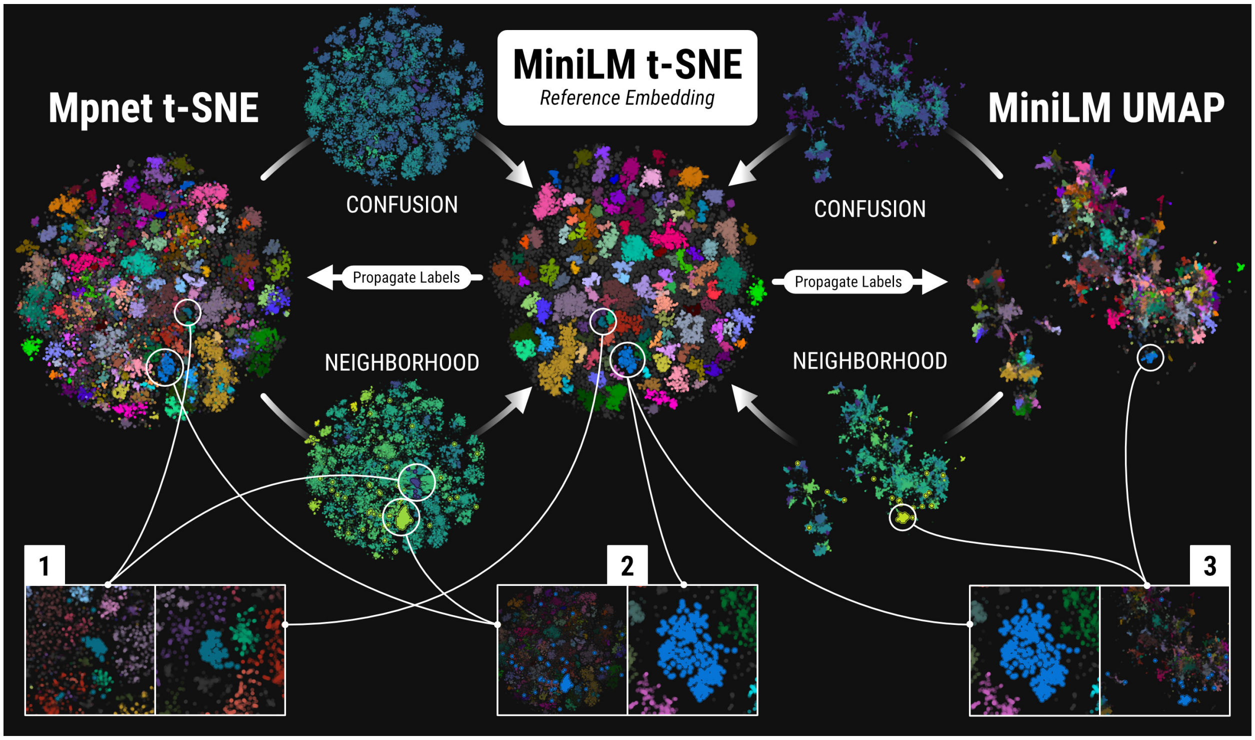

Figure S2: **Comparing News Articles** Middle: The reference embedding that was constructed with `minilm` plus t-SNE and used for clustering the points. The clusters are visualized by the point color where dark gray refers to points considered noise. Left: A second embedding constructed with `mpnet` plus t-SNE. To compare the two embedding methods, we propagated the point labels from the middle given one-to-one point correspondences. Right: A third embedding constructed with `minilm` plus UMAP. In between the left and middle as well as middle and right, we show four additional plots that visualize the Confusion (top) and Neighborhood (bottom) metrics. All metrics are computed in contrast to the reference embedding in the middle.

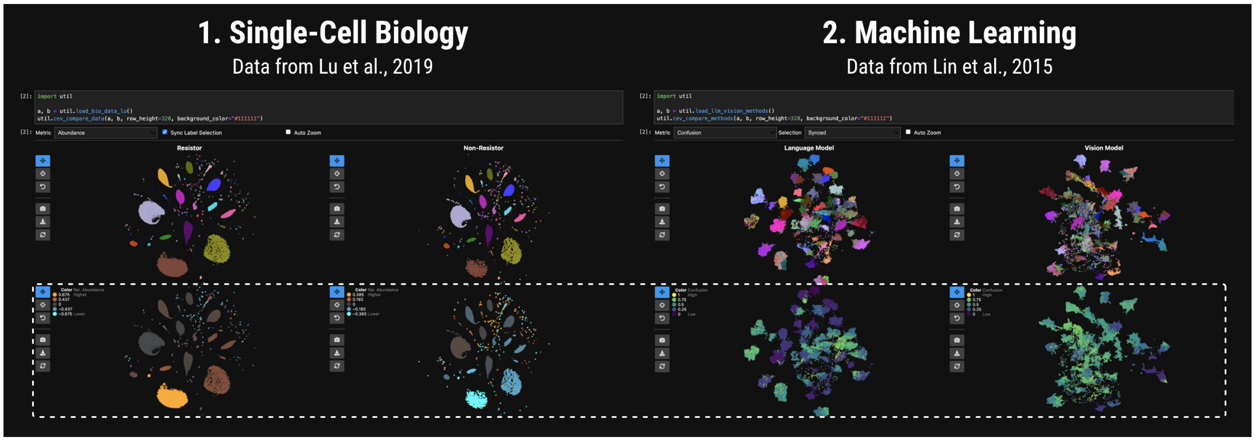

Figure S3: **User Study Interface.** Participants compared two embeddings using our interactive Jupyter widget prototype. This figures shows two example datasets: a single-cell biology dataset from Lu et al., [9] and the COCO dataset [3]. The second row of scatter plots that visualize our metrics is only shown in tasks 2A and 2B.

References

[1] L. van der Maaten and G. Hinton, “Visualizing data using t-SNE,” *J. Mach. Learn. Res.*, 2008.

[2] L. McInnes, J. Healy, and J. Melville, “Umap: Uniform manifold approximation and projection for dimension reduction,” 2020.

[3] T.-Y. Lin, M. Maire, S. Belongie, J. Hays, P. Perona, D. Ramanan, P. Doll ár, and C. L. Zitnick, “Microsoft COCO: Common objects in context,” in *Computer Vision–ECCV 2014: 13th European Conference, Zurich, Switzerland, September 6-12, 2014, Proceedings, Part V 13*. Springer, 2014, pp. 740–755.

[4] A. Dosovitskiy, L. Beyer, A. Kolesnikov, D. Weissenborn, X. Zhai, T. Unterthiner, M. Dehghani, M. Minderer, G. Heigold, S. Gelly, J. Uszkoreit, and N. Houlsby, “An image is worth 16x16 words: Transformers for image recognition at scale,” *ICLR*, 2021.

[5] N. Reimers, “Sentence transformer: all_minilm_l6_v2,” https://huggingface.co/sentence-transformers/all-MiniLM-L6-v2, accessed: 2023-09-12.

[6] F. Mair, J. R. Erickson, M. Frutoso, A. J. Konecny, E. Greene, V. Voillet, N. J. Maurice, A. Rongvaux, D. Dixon, B. Barber *et al.*, “Extricating human tumour immune alterations from tissue inflammation,” *Nature*, vol. 605, no. 7911, pp. 728–735, 2022.

[7] E. Greene, G. Finak, L. A. D’Amico, N. Bhardwaj, C. D. Church, C. Morishima, N. Ramchurren, J. M. Taube, P. T. Nghiem, M. A. Cheever, S. P. Fling, and R. Gottardo, “New interpretable machine-learning method for single-cell data reveals correlates of clinical response to cancer immunotherapy,” *Patterns (N Y)*, vol. 2, no. 12, p. 100372, Dec. 2021.

[8] E. Greene, G. Finak, F. Lekschas, M. Smith, L. A. D’Amico, N. Bhardwaj, C. D. Church, C. Morishima, N. Ramchurren, J. M. Taube, P. T. Nghiem, M. A. Cheever, S. P. Fling, and R. Gottardo, “Data Transformations for Effective Visualization of Single-Cell Embeddings,” Jul. 2022, version 1.0.0. Available at: https://github.com/flekschas-ozette/ismb-biovis-2022. DOI: 10.5281/zenodo.7522322. License: Apache-2.0.

[9] L. L. Lu, M. T. Smith, K. K. Yu, C. Luedemann, T. J. Suscovich, P. S. Grace, A. Cain, W. H. Yu, T. R. McKitrick, D. Lauffenburger *et al.*, “Ifn-γ-independent immune markers of mycobacterium tuberculosis exposure,” *Nature medicine*, vol. 25, no. 6, pp. 977–987, 2019.
